# Open field study on the efficacy of fluralaner topical solution for long-term control of flea bite allergy dermatitis in client owned cats in Ile-de-France region

**DOI:** 10.1186/s12917-019-2081-8

**Published:** 2019-10-11

**Authors:** Amaury Briand, Noelle Cochet-Faivre, Pascal Prélaud, Rob Armstrong, Céline Hubinois

**Affiliations:** 1 Department of Parasitology, Mycology, Dermatology, EA 7380 UPEC Dynamyc, Ecole nationale vétérinaire d’Alfort, F-94700 Maisons-Alfort, France; 2 ADVETIA Veterinary Hospital center, 9 Avenue Louis Breguet, 78140 Vélizy-Villacoublay, France; 3 Merck Animal Health, 2 Giralda Farms, Madison, NJ USA; 4 MSD Santé Animale, 7 Rue Olivier de Serres - Angers Technopole CS17144 -49 071, Beaucouze, France

**Keywords:** Fleas, Cat, Allergic dermatitis, Fluralaner, Clinical score, Ectoparasite

## Abstract

**Background:**

Flea bite is considered to be the main cause of allergic dermatitis in cats. There is a need for treatments able to control clinical signs of allergic dermatitis associated with flea bite in cats.

This was an open pre-treatment versus post-treatment clinical field study. All cats included in the study presented pruritus, skin lesions or other evidence compatible with flea infestation. Skin lesions were assessed (using SCORFAD) at days 0, 28, 56 and 84 whereas pruritus severity was assessed (using PVAS) at days 0, 15, 28, 56 and 84. On day 0, The fluralaner (280 mg/ml) product (Bravecto® spot-on for cats) was supplied in pipettes containing 0.4, 0.89 and 1.79 ml for cats of 1.2–2.8 kg, > 2.8–6.25 kg and > 6.25–12.5 kg body weight, respectively. The other animals living in the same household also received fluralaner. Based on cytological examination at day 0, oral amoxicillin and clavulanic acid was prescribed for 21 days if indicated. For cats presenting intense pruritus and discomfort at day 0, oral prednisolone at 0.5 mg/kg was prescribed for 3 days.

**Results:**

During the study all cats, except for one (cat number 10), improved significantly. Post-treatment median SCORFAD scores at all evaluations were significantly different from the pre-treatment score on day 0 (*P* values < 0.002 for all three post treatment examination days) with a score reduction of 49% on day 28, 79% on day 56 and 87% on day 84. The PVAS score decreased significantly over the study period for all cats but one (cat number 10). Post-treatment median PVAS scores at all evaluations were significantly different from the pre-treatment PVAS score on day 0 (*P* value < 0.002 for all four post-treatment days) with a reduction of 46% on day 15, 67% on day 28, 82% on day 56 and 92% on day 84. No adverse reaction or other health issue was reported during the study.

**Conclusions:**

A single topical treatment with fluralaner results in a significant reduction of flea bite allergic dermatitis clinical signs in cats over the subsequent 12 weeks without any additional environmental treatment.

## Background

Flea bite is considered to be the main cause of allergic dermatitis in cats and is a consequence of exposure to the ectoparasitic species *Ctenocephalides felis felis*. Clinical signs are not pathognomonic and usually consist of the pruritic papular dermatitis commonly known as feline miliary dermatitis. The associated pruritus varies in intensity and can lead to excoriation and self-induced alopecia. Eosinophilic granuloma complex lesions can also develop. In some cases, self-induced trauma can lead to secondary pyoderma [1]. Rapid and effective treatment is necessary to alleviate pruritus and resolve clinical signs. A multimodal medical approach may be necessary with administration of an antiparasitic agent, anti-pruritic treatment and antibiotics if intense pruritus or secondary infections are present [1].

The isoxazolines are a new class of ectoparasiticides comprising four commercially available molecules: afoxolaner, fluralaner, lotilaner and sarolaner, although one of these (afoxolaner) is not approved for use in cats. These compounds are active against γ-aminobutyric acid-(GABA) and glutamate gated chloride channels with significant selectivity for the insect neuron receptor over mammalian ones [2].

A fluralaner spot-on (Bravecto Spot-On, MSD Santé Animale, Beaucouzé, France) formulation was recently approved for treating dogs and cats. This treatment provides at least 12 weeks of flea and tick control after a single dose with a rapid onset of action, achieving 100% efficacy against fleas within 12 h of initial administration to cats [3]. Several controlled field studies have shown the safety and efficacy of topical fluralaner for feline flea infestation control with more than 99% flea count reduction over the 12-week recommended treatment interval [4, 5].

The aim of this field study was to assess the efficacy of fluralaner topical solution for the treatment of clinical signs compatible with flea bite allergic dermatitis in client owned cats maintained in private households.

## Results

Thirteen privately owned cats were included in this field study (Table 1). Cats were between 6 months and 18 years old and weighing 2.8 to 7.3 kg. They all lived indoors, with 4 having limited access to a garden. All cats included in the study were of European breed. Two cats had another companion cat in the household. At day 0, clinical signs included self-induced alopecia (85% of the cats), miliary dermatitis (77%), excoriations (70%) and eosinophilic granuloma complex (30%). Six cats presented two types of lesions, five cats presented three types of lesions and two cats presented four types of lesions (Table 1).

**Table 1:** Population characteristics and clinical presentation for cats included in a field study of Flea Allergy Dermatitis

Case number	Age (years)	Weight (kg)	Sex^a^	Type of lesion^b^	Sign of flea infestation^c^	Presence of fleas/flea feces at day 0	Concomitant treatment(s)^d^
1	0.6	4.2	M	EP, SA	I- infestation in the last year	No. Pulicosis at 2mth old. No treatment since July.	Pred: 0.5 mg/kg/d 3 days
2	11	5.9	MC	Exc, EP, MD, SA	I- infestation in the last year	Fleas seen by owner 5mths ago. One topical treatment.	ACA: 15 mg/kg/12 h 21 day. Pred: 0.5 mg/kg/d 3 days
3	12	3.7	FC	MD, SA	D- flea faeces	Flea faeces	No
4	9	5.5	MC	MD, SA, EP	D- fleas & flea faeces	Fleas (*n* = 1) and flea faeces	No
5	15	3.9	FC	Exc, MD, SA	D- fleas & flea faeces	Fleas (*n* = 2)	Pred: 0.5 mg/kg/d 3 days
6	18	3.7	MC	MD, SA	D- flea faeces	Flea faeces	No
7	10	4.2	FC	Exc, MD, SA	I- infestation in the last year	No. Similar episode last year with fleas and flea faeces.	Pred: 0.5 mg/kg/d 3 days
8	5	9	M	Exc, MD	D- fleas seen by owner when combing	Fleas seen by owner when combing.	No
9	0.5	2.8	F	Exc, EP, MD, SA	I-infestation 2 months ago	Fleas suspected by owner 2 mths ago. Treatment with essentiel oil 2 mths ago.	No
10	4	4.3	FC	EP, SA	I- infestation in the last year	No. Similar episode last year with fleas found by veterinarian.	ACA: 15 mg/kg/12 h 21 day. Pred: 0.5 mg/kg/d 3 days
11	10	7.2	MC	Exc, SA	D- fleas seen by owner when combing	Fleas seen by owner when combing.	Pred: 0.5 mg/kg/d 3 days
12	4	4.2	M	Exc, MD	D- flea faeces	Flea faeces	No
13	12	7.3	MC	Exc, MD, SA	D- fleas & flea faeces	Fleas (*n* = 1) and flea faeces	Pred: 0.5 mg/kg/d 3 days

^a^*M* male, *MC* male castrated, *F* female, *FC* female castrated

^b^*EP* eosinophilic plaque, *SA* self-induced alopecia, *Exc* excoriation, *MD* miliary dermatitis

^c^*I* indirect sign, *D* direct sign

^d^*Pred* prednisolone, *ACA* amoxicillin clavulanic acid

During the first consultation (day 0), eight cats presented with direct signs of fleas: fleas and flea feces were observed on three cats; flea feces only were observed on three other cats; and two owners reported to have seen fleas on their cat while combing them the day before the consultation. For the remaining five cats, indirect signs of flea infestation were reported including a history of flea infestation with similar signs and presence of fleas reported in the previous year without adequate treatment.

All 13 study cats (and the two companion cats) were treated with one topical dose of fluralaner on day 0. No antiparasitic sprays or foggers were used in the home to eliminate flea adults or immature stages in the environment. Six cats received no other concomitant treatment. Two cats were treated with oral amoxicillin clavulanic acid for secondary pyoderma based on cytological examination, and seven cats received prednisolone for pruritus (Table 1).

**Fig. 1:**
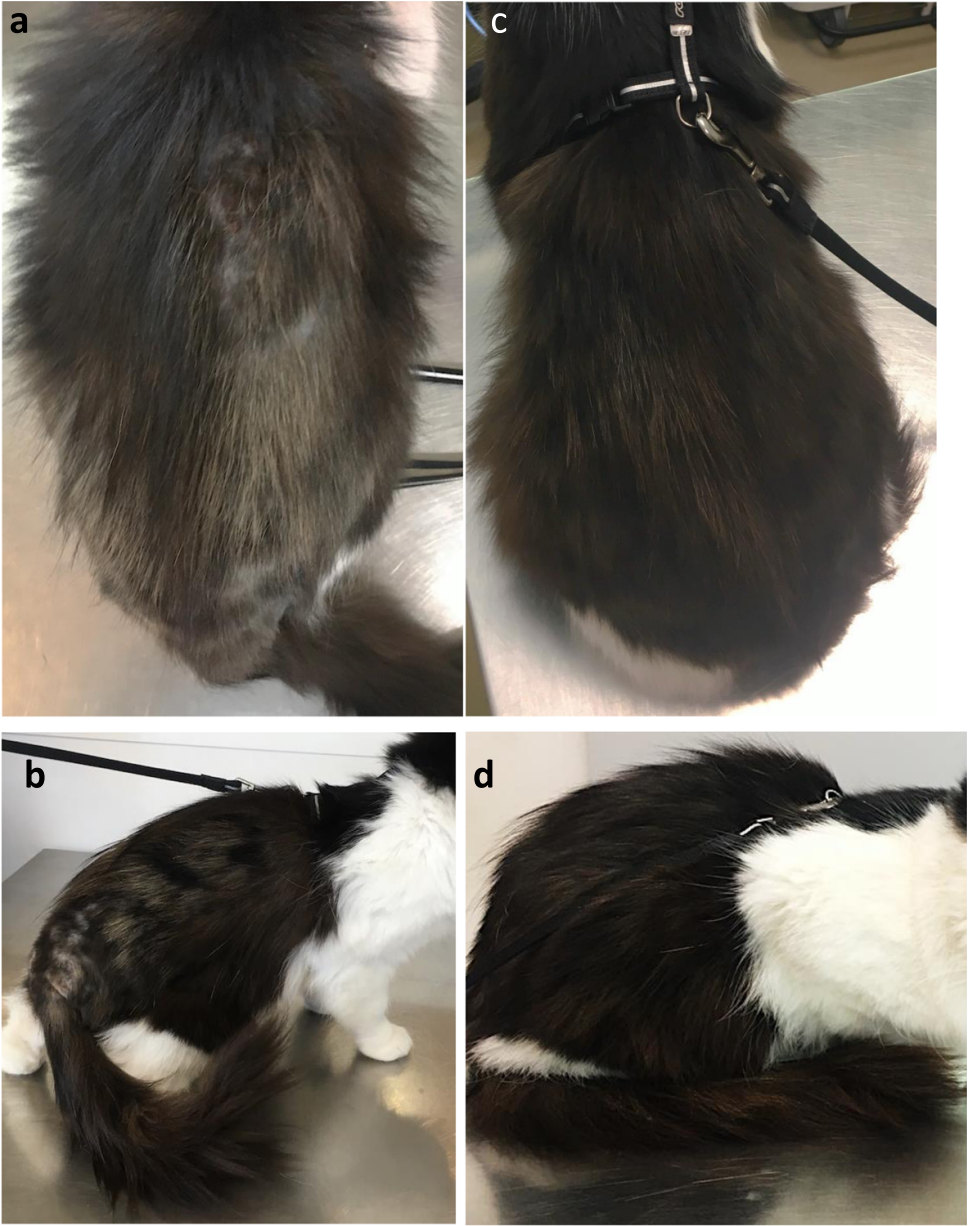
10-year-old European cat presented with skin lesions on the dorsolumbar area and the flanks on day 0 (**a** & **b**) and the same cat on day 84 (**c** & **d**), showing complete lesion resolution

All cats were flea free at day 28, 56 and 84. Clinical scores (SCORFAD score) at day 0 ranged between 5 and 10 with a median of 7. During the study all cats improved significantly (Table 2, Fig. 1) except cat 10. Post-treatment median SCORFAD scores at all evaluations were significantly different from the pre-treatment score on day 0 (*P* values < 0.002 for all three post treatment days) with a score reduction of 49% on day 28, 79% on day 56 and 87% on day 84. Owner assessed pruritus scores (PVAS score) ranged between 4 and 10 at day 0 with a median of 8.3. The PVAS score decreased significantly over the study period for all cats (Table 2) except cat 10. Post-treatment median PVAS scores at all evaluations were significantly different from the pre-treatment PVAS score on day 0 (*P* value < 0.002 for all four post-treatment days) with a reduction of 46% on day 15, 67% on day 28, 82% on day 56 and 92% on day 84.

**Table 2:** Evolution of clinical score (SCORFAD) and pruritus score (PVAS) in cats following topical fluralaner treatment

Parameter	Day 0	Day 15	Day 28	Day 56	Day 84
Median SCORFAD	7		3	1	0
Median PVAS	8.3	3.8	2.5	1	0
*P* value compared with Day 0		< 0.002	< 0.004	< 0.002	< 0.002

The PVAS and SCORFAD scores were not significantly different between cats with or without direct evidence of fleas at day 84 (Table 3). The median PVAS scores improvement was not significantly different between cats that did or did not get prednisolone at day 84 whereas the median SCORFAD scores improvement was significantly lower for cats that received a short course of prednisolone (Table 4). No adverse reaction or other health issue was reported for any fluralaner treated cat at any time point during the duration of the study.

**Table 3:** Comparison of percentage of improvement of SCORFAD and PVAS scores between day 0 and 87 for cats with direct signs of flea infestation and cats with indirect signs of flea infestation

	Cats with direct flea evidence	Cats with no direct flea evidence	*P* value
Median of PVAS improvement (%) at day 84	100	100	0.32
Median of SCORFAD improvement (%) at day 84	100	87.5	0.11

**Table 4:** Comparison of percentage of improvement of SCORFAD and PVAS scores between Day 0 and Day 87 for cats with or without treatment with prednisolone

	Cats with prednisolone	Cats without prednisolone	*P* value
Median of PVAS improvement (%) at day 84	100	100	0.10
Median of SCORFAD improvement (%) at day 84	88.9	100	0.018

## Discussion

This open field study demonstrates the value of a single treatment with topical fluralaner for control of clinical signs in cats associated with suspected FAD over the subsequent 12-week period. The clinical score (SCORFAD) and PVAS reduction observed 12 weeks after treatment was similar to the reduction observed in a recent field study on the efficacy of fluralaner topical solution to treat flea infestation in cats [5]. This previous study focused on flea count reduction following treatment and assessed changes in clinical signs in cats; the presence of flea was the principal inclusion criteria whereas the clinical score and pruritus were not considered [5]. The study reported here used the SCORFAD score, which is validated for assessment of clinical le7sion in cats suffering from allergic dermatitis [6], because clinical evaluation can be difficult in the cat. Pruritus assessment can also be difficult in the cat, and the PVAS scoring system used in this study was directly adapted from the analogous PVAS scoring system used to assess pruritus in dogs [7].

Cats included in this field study presented signs compatible with flea bite allergy dermatitis with either indirect signs of flea infestation reported by the owners or direct visualization of fleas or flea feces. The dramatic improvement in clinical signs observed over the 12-week treatment period in 12 of 13 cats is further confirmation that these cats suffered from flea bite allergic dermatitis.

One cat (case 10, Table 1) showed no improvement in either pruritus or clinical signs during the study period. This cat did show initial partial improvement with prednisolone and oral amoxicillin and clavulanic acid, and quickly relapse after discontinuation of therapy. Cat 10 had no direct signs of flea infestation and it may have suffered from a different allergy than to flea bites.

Flea absence was not an exclusion criterion for study enrolment and previous studies showed that cats with flea allergy dermatitis can present with either very few or no fleas on clinical examination [8]. This could be because the high level of pruritus in flea bite allergic cats leads to over grooming and removal of more fleas compared to unaffected cats. Examination of cats in an indoor colony found that those suffering from flea bite allergy harbored an average of 0.75 fleas whereas healthy cats had an average of 5.2 fleas. Some of the flea bite allergy affected cats were found to have no fleas [8]. This result confirmed that absence of fleas or a low level of flea infestation does not rule out flea bite allergic dermatitis.

Seven cats in this study received a short course (3 days) of prednisolone starting on day 0 to alleviate the discomfort of intense pruritus (Table 1). Prednisolone administration was recommended because a short (3 day) course of oral prednisolone at this dosage would not likely alter the results of the 3 month-study at subsequent time points while providing immediate relief and respecting the cat’s welfare. The prednisolone biological half-life in the cat is reported to be similar to the human half-life of between 12 and 36 h [9]. Three days of prednisolone at this low dose was not expected to have an impact on the clinical evolution of these cases. However, the median improvement of SCORFAD scores in these seven prednisolone treated cats was significantly lower at day 84. This result may have occurred because concomitant treatment was given at enrolment to cats with more intense pruritus and more severe clinical signs. Alternatively, the median SCORFAD score improvement may have remained lower in these prednisolone treated cats (although improvement was noted) if they suffered from more than one allergy i.e. some improvement in their scores was seen following flea treatment but their score improvement was lower because of another continuing allergy.

Starting with the first post treatment clinical examination on day 28, no further fleas or flea feces were found on any cat; and although pruritus and clinical scores continued to decrease after this time they did not drop immediately. This time required for clinical sign recovery could be related to the persistence of skin inflammation beyond the removal of the inciting parasites. In addition, secondary infections and chronic changes associated with skin lesions and self-trauma require time to resolve. Fluralaner is a very effective flea adulticide but does not kill the non feeding immature stages and it is likely that over the initial weeks following treatment, immature flea stages in the household continued to mature into adults, could take a first feeding and that trigger inflammatory reactions which can explain continuing clinical signs. This effect depends on the immature stage load in the household and also on the climatic conditions which play a role in the speed of development of immature stages into adult fleas.

Fluralaner has a rapid onset of action against fleas with a proven high level of efficacy over the subsequent 12 weeks even with constant parasitic challenge [3, 10]. This 12-week duration of efficacy leads to a major improvement in owner compliance for dogs [11] and this is likely also true for cats. The convenience of 12 weeks of efficacy may be even more critical for cats because they can be more difficult for owners to treat. The extended efficacy duration simplifies treatment administration and also, as shown in this study, cats with flea bite allergy can recover without additional treatment of the environment like insecticide foggers or sprays. Poor owner compliance with treatments that have a shorter efficacy duration can result in a failure to control flea infestations because juvenile fleas continue to mature into reproducing adults and repopulate the home when the treatment schedule is not maintained [12].

## Conclusion

In conclusion, this field study showed that a single topical treatment with fluralaner results in a significant reduction in flea bite allergic dermatitis clinical signs in cats over the subsequent 12 weeks without any additional environmental treatment.

## Methods

This was an open pre-treatment versus post-treatment clinical field study. Cats were presented to the veterinary dermatology service between December 2016 and December 2018. All cats were from the Ile-de-France region and were included in the study with a written informed consent from the owner. The study was conducted according to good clinical practice and animal welfare principles.

Enrolled cats were kept at home in their usual condition and fed their usual diet during the study. All cats included in the study presented with pruritus, skin lesions or other evidence compatible with flea infestation. Evidence of flea infestation was defined as: presence of at least one flea or flea feces noted by the examining veterinarian, fleas noted by the owner during the days prior to the consultation or a previous flea infestation episode reported by the owner in the year before presentation. Cats presented pruritus or the following clinical signs compatible with allergic dermatitis: miliary dermatitis, excoriations, self-induced alopecia and eosinophilic granuloma complex. Exclusion criteria were: cats less than 11 weeks old or less than 1.2 kg according to the summary of product characteristics, cats treated with an antiparasitic treatment during the month before inclusion, cats treated within 7 days with an antihistamine, cats treated within 1 month with an oral corticosteroid or oral cyclosporine, or cats treated within 60 days with an injectable corticosteroid. When indicated by the clinical signs, cats had a skin cytological exam for signs of secondary pyoderma and a skin scraping or fungal culture to exclude other infections or parasitic disease.

The study period for each cat was 84 days and included 4 visits: day 0, day 28, day 56 and day 84. During the first visit on day 0 the patient history was recorded and a general clinical evaluation performed. The presence of pruritus or dermatological signs compatible with allergic dermatitis were noted. Two scoring systems, “Scoring Feline Allergic Dermatitis” (SCORFAD) and “pruritus visual analogue scale” (PVAS), were used to measure clinical signs and pruritus. The SCORFAD method was specifically developed and validated to assess the severity and extent of four lesions (excoriation, miliary dermatitis, self-induced alopecia and eosinophilic plaque) including the number of body regions involved. Each of the four lesions was assigned a score from 0 to 4 leading to a maximum score of 16 [6]. The owner assessed the pruritus severity using a PVAS. This non-numeric scale was a modification of a previous published feline pruritus score [6]. Briefly, descriptions of increasing pruritus severity are provided on a non-numeric scale and the owner rates the level of pruritus using this scale. This rating was then converted to a numerical value as previously described for the dog [13]. Skin lesions (SCORFAD) were assessed at day 0, 28, 56 and 84 whereas pruritus severity was assessed at day 0, 15, 28, 56 and 84. When fleas were observed on physical examination, they were counted by combing the cat for at least 5 min until no further fleas were found over three consecutive minutes of combing.

On day 0, each cat was treated with a topical application of fluralaner at the commercial dose based on the cat’s weight. The fluralaner (280 mg/ml) medication (Bravecto® spot-on for cats) was provided in pipettes containing 0.4, 0.89 and 1.79 ml for cats weighing 1.2–2.8 kg, > 2.8–6.25 kg and > 6.25–12.5 kg respectively. Treatment was applied on the skin at the base of the skull and of cats on a single occasion at Day 0, at a dose rate of 40–94 mg fluralaner body weight [14].

Other animals living in the same household also received a fluralaner topical treatment (Bravecto® Spot-On for cat) or oral fluralaner (Bravecto® Chew for dogs). Based on skin cytological examination at day 0, oral amoxicillin and clavulanic acid was prescribed for 21 days if indicated. For cats presenting with intense pruritus and discomfort at day 0, oral prednisolone at 0.5 mg/kg was prescribed for 3 days.

At each time point, t, clinical signs (or pruritus severity) reduction was calculated using the following formula:

Clinical signs (or pruritus severity) reduction (%) = 100 × (mean day 0 – mean t)/mean day 0.

For time point comparison of SCORFAD and PVAS a paired Wilcoxon test was used. Comparison of the PVAS and SCORFAD at day 84 between cats with direct or indirect signs of flea infection and between cat with or without concomitant treatment was performed with a Mann Whitney test. Statistical significance was declared when *P* <  0.05. Statistical tests were performed using a website dedicated to statistical analysis (BiostaTGV, http://marne.u707.jussieu.fr/biostatgv/).

## Data Availability

Data from this clinical study are proprietary and maintained by MSD Santé Animale. Data will be shared with qualified investigators on contact and review of the qualifications and specific request. Please contact Céline Hubinois: MSD Santé Animale, 7 Rue Olivier de Serres - Angers Technopole CS17144–49 071 BEAUCOUZE, France. celine.hubinois@msd.com

## Abbreviations

GABA: γ-aminobutyric acid
PVAS: Pruritus visual analogue scale
SCORFAD: Scoring Feline Allergic Dermatitis

## References

[1] Miller WH, Griffin CE, Campbell KL, Miller WH, Griffin CE, Campbell KL (2013). Feline fleabite hypersensitivity. Muller & Kirk’s small animal dermatology.

[2] Gassel M, Wolf C, Noack S, Williams H, Ilg T (2014). The novel isoxazoline ectoparasiticide fluralaner: selective inhibition of arthropod γ-aminobutyric acid- and L-glutamate-gated chloride channels and insecticidal/acaricidal activity. Insect Biochem Mol Biol.

[3] Dongus H. Bravecto® für die Katze: Innovative Zecken- und Flohbekämpfung mit 12 Wochen Wirksamkeit. Sonderdruck Aus Kleintiermedizin. 2016;1:196–8.

[4] Meadows C, Guerino F, Sun F (2017). A randomized, blinded, controlled USA field study to assess the use of fluralaner topical solution in controlling feline flea infestations. Parasit Vectors.

[5] Dryden MW, Canfield MS, Bocon C, Phan L, Niedfeldt E, Kinnon A (2018). In-home assessment of either topical fluralaner or topical selamectin for flea control in naturally infested cats in West Central Florida, USA. Parasit Vectors.

[6] Steffan J, Olivry T, Forster SL (2012). Responsiveness and validity of the SCORFAD, an extent and severity scale for feline hyper-sensitivity dermatitis. Vet Dermatol.

[7] Crosaz O, Chapelle E, Cochet-Faivre N, Ka D, Hubinois C, Guillot J (2016). Open field study on the efficacy of oral fluralaner for long-term control of flea allergy dermatitis in client-owned dogs in Ile-de-France region. Parasit Vectors.

[8] Cadiergues M-C, Pressanti C (2014). Efficacy of Spinosad tablets administered to a colony of 15 indoor cats naturally infested with fleas.

[9] Lowe AD, Campbell KL, Graves T (2008). Glucocorticoids in the cat. Vet Dermatol.

[10] Ranjan S, Young D, Sun F (2018). A single topical fluralaner application to cats and to dogs controls fleas for 12 weeks in a simulated home environment. Parasit Vectors.

[11] Lavan R, Armstrong R, Burgio F, Tunceli K (2018). Duration of annual canine flea and tick protection provided by dog owners in Spain. Parasit Vectors.

[12] Dryden MW, Smith V, Bennett T, Math L, Kallman J, Heaney K, Sun F (2015). Efficacy of fluralaner flavored chews (Bravecto) administered to dogs against the adult cat flea, Ctenocephalides felis felis and egg production. Parasit Vectors.

[13] Rybnícek J, Lau-Gillard PJ, Harvey R, Hill PB (2009). Further validation of a pruritus severity scale for use in dogs. Vet Dermatol.

[14] Rohdich N, Zschiesche E, Wolf O, Loehlein W, Pobel T, José Gil M, Roepke KA (2018). Field effectiveness and safety of fluralaner plus moxidectin (Bravecto® plus) against ticks and fleas: a European randomized, blinded, multicenter field study in naturally-infested client-owned cats. Parasit Vectors.

